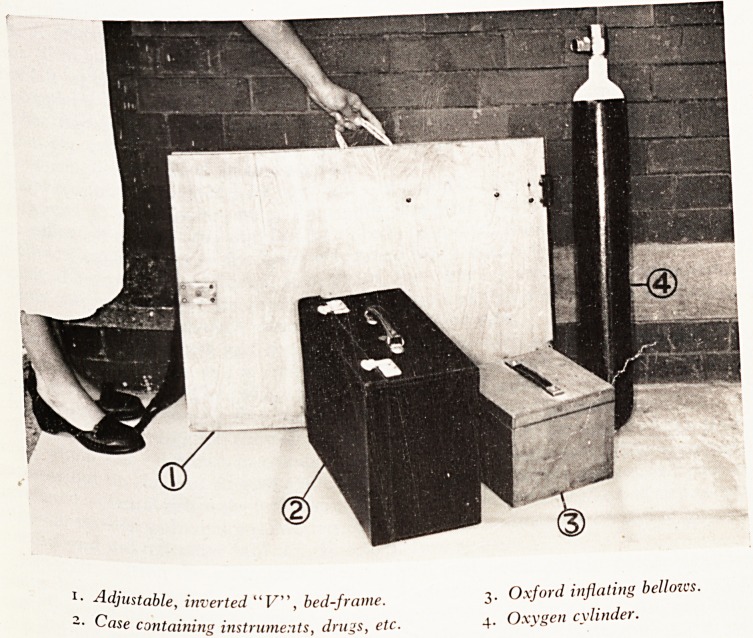# Transporting Severely Paralysed Patients

**Published:** 1959-04

**Authors:** James Macrae

**Affiliations:** Ham Green Hospital, Bristol


### TRANSPORTING SEVERELY PARALYSED PATIENTS

BY

JAMES MACRAE, M.D.

*Ham Green Hospital, Bristol*

The transport of patients suffering from diseases which impair breathing or swallowing, or both, is a problem which has become of great importance during the past decade. This is because most of these patients suffer from poliomyelitis, and the recently established respiratory centres now provide a much better service and are able to save the lives of many of these desperately ill patients. The spectacle of a racing ambulance carrying a patient whose unassisted vital capacity is ebbing is now an unnecessary drama. During the years 1947 to 1954 there were nine such wild drives which ended in this hospital with a dead patient: one of these, a young man from Bridgwater appeared to have died in the ambulance a mile away; a boy of sixteen died in his own home six miles from Ham Green before the arrival of an ambulance which was stationed at Weston-super-mare. During these years only one patient was transferred to us from another hospital under conditions of planned transport. She arrived in a tank respirator driven by a precarious system of batteries and transformers. She was accompanied by eight men and two nurses; everything and everybody travelled in a furniture van. After such a valiant effort it was a pity that the patient inspired vomit during the move and died as a result within the day.

With the introduction of artificial positive pressure respiration in 1953 and an increasing experience of the management of cases suffering from respiratory insufficiency, a better and safer method of transport was obviously necessary. Thus, in 1955, we thought it a duty of this hospital to provide a service designed to get these patients safely into our respiratory unit.

Initially the plan seemed to have three elements:

(*a*) The reporting of cases;

(*b*) Sending assistance and equipment to the patient;

(*c*) Transporting the patient to Ham Green.

We imagined that this hospital would provide doctors, nurses and equipment ready to go to the patient at short notice and we thought we might need to do tracheotomy, set up intravenous drips, give nasal-gastric tube feeds and possibly nurse patients at home or in another hospital for a few days before transfer was possible. Equipment in accordance with these ideas was assembled and proved to be very bulky. The provision of a specially equipped ambulance was mooted but the idea was discarded as impracticable. From the beginning, we decided to depend on manual methods of artificial breathing and so have never attempted to carry mechanical respirators for emergency cases.

The availability of this new transport service was advertised to Medical Officers of Health by the Regional Hospital Board and it was specially requested that the doctor in charge of the case should get in direct personal touch with doctors in this hospital whenever the service was required. It was considered that we would need a discussion with the doctor in charge in order to assess the needs of the case and to give advice which he could apply in maintaining the patient until the team from Ham Green could arrive. Undoubtedly this telephone conversation has remained the most important item of the emergency service.

Our first call for assistance came from Exeter on an August Bank holiday afternoon. The information received by telephone suggested a severe case of bulbar poliomyelitis, very nearly dead already. Hence, since our team could hardly get to Exeter in less than three or four hours, it was suggested that a local anaesthetist be called to insert an endo-tracheal cuffed tube quickly and breathe the patient manually on the journey t Ham Green. This was in fact done very successfully and the patient arrived in re markably good condition so that we could safely perform tracheotomy and maintai ventilation by a mechanical positive pressure machine. This first experience pointe to a fresh conception of the problem of transporting such patients: supervision an management should be undertaken by a local anaesthetist.

The hospital team is still in being; there are two doctors in this team without nurse this has proved quite sufficient. We discovered that the amount of equipment can b much reduced. What is required for these patients consists of:

(*a*) Equipment for posture in an ambulance, so we carry a simple, adjustabl inverted V frame for cases of simple swallowing defect;

(*b*) Equipment for manual mask ventilation for cases of breathing insufficiency onl

(*c*) Equipment for passing a cuffed endotracheal tube and manual ventilation there after for combined swallowing and breathing deficiency.

A limited assortment of drugs and instruments are carried, especially pentothal an scoline with sterilized syringes. A sucker and oxygen cylinder completes the equip ment. In all, these materials weigh about fifty pounds and are easily carried by han The sole purpose of the team is now confined to the reasonably quick movement o the patient to hospital, and the providing of adequate artificial respiration on the wa

Some twenty-six patients have actually been transported into hospital since 195 Nineteen have been cases of poliomyelitis; five were cases of infective polyneurit one of acute ascending myelitis and one with myasthenia gravis. Apart from o child with pneumonia we have not been sent any unsuitable cases. These patien have come from Exeter, Truro, Bideford, Taunton, Yeovil, Cheltenham, Glouceste Swindon, Bath and Bristol. In eight cases our own team went out to collect the patie and in all of the others we enlisted the aid of a local anaesthetist. The help th these anaesthetists have given has been invaluable, although as they themselves ha commented the job required is what they have to do every day—"breathe" a patie by hand through a mask or through an endotracheal tube. In some cases this assiste ventilation was unnecessary, but the anaesthetist was there in the ambulance to he if required. It has been found that once the patient's airway is clear and manu ventilation established, the patient is safe and can be transported long distanc without incident. The journey can be quite unhurried; there is no need for a speciall equipped ambulance, and suction is seldom required if the patient's airway is reason ably dry at the beginning of the journey.

One of the advantages of the initial telephone talk with the doctor in charge of th case, has been that a decision can be made whether a local anaesthetist should b called in. Then a short talk with the anaesthetist has always resulted in him under standing completely what is required so that the patient is brought to us competent and without fuss.

The results have been very good. There have been no deaths in an ambulance since the service started and all the patients transported have arrived in hospital in goo condition.

We employ the Oxford Inflating Bellows as a method of hand ventilation, using i either through a face mask or through an endotracheal tube. It is a most efficie instrument which only needs the addition of a pressure gauge in order to avoid to great ventilation pressures.

PLATE XXI

1. *Adjustable, inverted "V", bed-frame.*

2. *Case containing instruments, drugs, etc.*

3. *Oxford inflating bellows.*

4. *Oxygen cylinder.*

## Figures and Tables

**Figure f1:**